# Growing Problem of Multidrug-Resistant Enteric Pathogens in Africa

**DOI:** 10.3201/eid1311.070674

**Published:** 2007-11

**Authors:** Iruka N. Okeke, Oladiipo A. Aboderin, Denis K. Byarugaba, Kayode K. Ojo, Japheth A. Opintan

**Affiliations:** * Haverford College, Haverford, Pennsylvania, USA; † Obafemi Awolowo University, Ile-Ife, Nigeria; ‡ Faculty of Veterinary Medicine, Makerere University, Kampala, Uganda; § University of Washington, Seattle, Washington, USA; ¶ University of Ghana Medical School, Accra, Ghana

**Keywords:** enteric pathogens, antimicrobial resistance, antibiotic resistance, sub-Saharan Africa, perspective

## Abstract

Control of fecal–orally transmitted pathogens is inadequate in many developing countries, in particular, in sub-Saharan Africa. Acquired resistance to antimicrobial drugs is becoming more prevalent among *Vibrio cholerae*, *Salmonella enteritidis*, diarrheagenic *Escherichia coli,* and other pathogens in this region. The poor, who experience most of the infections caused by these organisms, bear the brunt of extended illness and exacerbated proportion of deaths brought about by resistance. Improved antimicrobial drug stewardship is an often cited, but inadequately implemented, intervention for resistance control. Resistance containment also requires improvements in infectious disease control, access to and quality assurance of antimicrobial agents, as well as diagnostic facilities. Structural improvements along these lines will also enhance disease prevention and control as well as rational antimicrobial drug use. Additionally, more research is needed to identify low-cost, high-impact interventions for resistance control.

## Abstract 2

A disproportionate number of low-income persons are affected.

The adverse effects of infectious diseases in many developing countries is considerable and, within those countries, economically disadvantaged persons are most likely to contract communicable diseases and least likely to access appropriate treatment (*1*,*2*). Many bacterial and parasitic diseases could, until recently, be treated with inexpensive antimicrobial agents, but treatment has recently been made more expensive and less successful by the emergence and spread of resistant organisms. Drug resistance is a large and growing problem in infections that account for most of Africa’s disease burden, including malaria, tuberculosis (TB), HIV infection, and respiratory and diarrheal diseases. The proportion of malaria infections resulting in death has increased in Africa, largely due to resistance, and the cost of effective antimalarial agents is higher than the health budgets of malaria-endemic countries can accommodate (*3*). Similarly, a recent outbreak of extensively drug-resistant TB in rural South Africa illustrated that resistant organisms pose an enormous and costly threat to HIV-infected persons and their HIV-negative contacts (*4*).

Much of the current discourse on infectious disease and drug resistance as it affects sub-Saharan Africa is limited to the pressing problems associated with HIV, TB, and malaria. Resistance, however, equally compromises the management of acute respiratory infections, sexually transmitted diseases, and diseases spread by the fecal–oral route, such as typhoid fever, cholera, dysentery, and other diarrheal diseases, which are the focus of this perspective. Moreover, young children are especially likely to acquire resistant enteric infections, from which they can experience less obvious, but long-term adverse effects.

## Increased Antimicrobial Drug Resistance in Enteric Bacteria

Cholera toxin–producing *Vibrio cholerae* cause the characteristic life-threatening gastroenteritis, cholera. At least 7 pandemics of the disease, originally designated “Asiatic cholera,” have occurred in recent history. The ongoing pandemic has seen the emergence of O139 *V. cholerae*, as a non-O1 pandemic strain and, importantly, the emergence and spread of drug-resistant O1 strains. The current focus of the cholera pandemic is Africa, which has seen two thirds of all cholera outbreaks in the last decade (*5*). The primary treatment for cholera is rehydration. Most patients will overcome the infection if they are rehydrated promptly and properly, even if they do not receive antimicrobial drugs. Antimicrobial drugs, however, shorten the course of infection and prevent person-to-person transmission, which may be crucial for slowing outbreaks because organisms from infected persons may be more virulent than those acquired in the wild (*6*). Antimicrobial agents may also be life-saving for malnourished and other immunocompromised patients who have cholera.

Tetracycline was the empiric drug of choice for cholera in Africa and elsewhere for many years. At the end of the 1970s, however, incompatibility group C tetracycline-resistant plasmids were isolated from *V. cholerae* isolates in Tanzania, Kenya, and other parts of Africa (*7*,*8*). In each case, resistance emerged during an ongoing epidemic where tetracycline was being used intensively for prophylaxis as well as treatment. Tetracycline has sequentially been replaced by trimethoprim-sulfamethoxazole and, more recently, quinolones, because of the emergence and spread of resistant strains. Molecular evaluation of more recent resistant *V. cholerae* isolates typically found regionally conserved plasmids, some of which carried class 1 integrons bearing multiple resistance cassettes (*9*,*10*). A chromosomally integrated transferable resistance element, SXT, has also spread worldwide and has been recently reported from Africa (*9*).

Emergence of resistance in *V. cholerae* has been linked to increased mortality rates in recent African outbreaks. Similar experiences have also been reported with *Shigella dysenteriae* type 1, another enteric pathogen that causes life-threatening disease and has epidemic potential. The impact of resistance in both pathogens is illustrated by an overwhelming outbreak in July 1994 at the Goma camp, which resulted in the deaths of ≈12,000 Rwandan refugees (*11*). More recently, Dalsgaard et al. (*12*) observed a marked increase in case-fatality rate during the 1997–1998 phase of a Guinea-Bissau cholera outbreak, compared to an overlapping 1996–1997 outbreak. A major feature of the latter wave of cholera was the presence of strains simultaneously resistant to ampicillin, erythromycin, tetracycline, furazolidone, aminoglycosides, trimethoprim, and sulfamethoxazole. These multidrug-resistant strains were not present during the first wave and probably arose following the acquisition of a 150-kb–pair resistance plasmid bearing a class 1 integron and genes encoding resistance to all antimicrobial agents commonly used in empiric management of cholera.

The emergence and spread of multidrug-resistant *Salmonella enterica* subsp. Typhi worldwide has had important consequences for mortality rates from typhoid fever (*13*). There are very few reports from Africa; nonetheless, available data suggest that although the problem may not be as intense as in other parts of the world, resistance has emerged, and alternatives to current treatment protocols are often not available or unaffordable. Multidrug-resistant nontyphoidal *Salmonella* spp. (NTS) have emerged as a global public health threat. In industrialized countries, they are most commonly associated with foodborne gastroenteritis. In parts of sub-Saharan Africa, however, NTS are important causes of life-threatening bacteremia. Studies from Kenya have found that community-acquired NTS are among the top 3 causes of death among children <5 years of age (*14*,*15*). Moreover, pulsed-field gel electrophoresis data suggest that most life-threatening disease is caused by isolates that are clonal in origin (*14*). In a recent study, children from poor slums of Kenya were significantly more likely to be infected with multidrug-resistant NTS than were children from middle-income families (*14*). The patterns of resistance among these strains suggest that third-generation cephalosporins should be the drug of choice for empiric management of these infections, but in most cases, these drugs are too expensive.

Antimicrobial drug resistance is a large and growing problem among organisms that cause diarrheal disease. Although most diarrheal diseases are self-resolving and should not be treated with antimicrobial agents, invasive or protracted infections require chemotherapy and are typically managed empirically. Recent data from Gabon, Kenya, Nigeria, Senegal, and Tanzania suggest that resistance among causative organisms of these infections, such as enterotoxigenic, enteropathogenic, and enteroaggregative *Escherichia coli,* is high and appears to be rising (*16*–*18*). Although oral rehydration therapy has drastically reduced deaths from the disease, prolonged infectious bouts of diarrhea have long-term consequences for physical and cognitive development. Very few reports have examined the epidemiology of diarrheal pathogens and even fewer have looked at drug resistance. Notable drug-resistant enteropathogenic *E. coli* outbreaks and sporadic cases have been reported from several African countries, including Kenya and Tanzania (*16*,*19*). The more recently defined enteroaggregative *E. coli* are typically multidrug-resistant and are one of the most common causes of childhood diarrhea, particularly persistent infections (*20*). Antimicrobial drug–resistant diarrheagenic *E. coli* pathotypes, including enteroaggregative *E. coli*, are also emerging as important diarrheal pathogens in AIDS patients (*21*).

Surveillance in healthy populations has demonstrated that commensals constitute a rich reservoir of genetic material from which pathogens can readily acquire resistance on mobile elements. A long-term study in Nigeria showed that resistance of commensal *E. coli* to almost all agents studied increased rapidly over time (*22*). Additionally, urban residents in Nigeria, Ghana, and Zimbabwe were more likely to carry multidrug-resistant *E. coli* than were rural or provincial residents (*23*,*24*). This finding has important consequences in light of the rapid rate of urbanization in these countries and other parts of the continent. Travel networks have become more efficient and are more extensively used. Therefore, just as Africa has had to deal with imported resistant organisms, resistant strains that emerge or are amplified in Africa will be exported (*25*,*26*).

Two overlapping problems are worsening the situation regarding diarrheal disease within Africa: the failure to control the spread of diarrheal pathogens, due to unclean water, poor sanitation, and malnutrition; and the failure to contain resistant organisms and resistant genes so that, when infections occur, they produce more adverse consequences. It is perhaps obvious, if unaddressed, that poor and displaced persons in Africa are least likely to be able to access potable water, safe sanitation, and other factors to prevent fecal–oral infection and that public health facilities need to be strengthened to protect the poor (*27*,*28*). However, the poor also disproportionately bear consequences from drug resistance, and interventions to curb the current trend are sorely needed. Economists describe a situation in which the decision to use a commodity that produces a deleterious byproduct that imposes costs on other persons, who are not part of the decision, as a negative externality. Because resistance is an externality, the poor are victims of the resistance-predisposing activities of the affluent, including the presence of visitors from other countries. Furthermore, resistance-promoting activities and the consequences of resistance more often than not occur at a different time and place. It is therefore unrealistic to expect that the poor will mount resistance-curbing interventions as a priority without prompting and support.

## Overcoming Roadblocks to Containing Drug Resistance in Africa

There are difficulties associated with monitoring resistance in many parts of Africa, but sufficient published data exist to suggest that resistance rates are high and rising (*22*,*29*). In 2001, the World Health Organization issued a strategy for resistance containment (*30*). Most developing countries, particularly those in sub-Saharan Africa, have yet to implement any of the recommended interventions in spite of a mounting resistance crisis. Special risk factors for resistance, and roadblocks for evaluating and implementing interventions, are linked to patient poverty and health system poverty. This is evident in sub-Saharan Africa, where we study resistance, but also in other developing countries.

Industrialized countries that are actively addressing community-acquired resistance have typically prioritized those interventions predicted to lower total antimicrobial agent consumption and therefore selective pressure (*31*,*32*). An important concern for poor countries is not the total amount of antimicrobial agents consumed, whose need exceeds the resources available, but the way these drugs are used. Diagnostic imprecision spurs overprescribing, particularly of broad-spectrum agents, and low antimicrobial diversity promotes emergence and spread of potentially epidemic resistant clones. In Africa, medicines are available from unorthodox sources and persons earning low daily wages often procure them 1 dose at a time. Sources include itinerant vendors, often encountered on buses in many African countries, who cannot always be located should a patient choose to purchase more doses later (Figure). Poor patients have little personal incentive to purchase more medicine than is needed to produce short-term relief. Additionally, regimen fragmentation comes without sanction and allows for large price mark-ups and consequent exploitation. Therefore, distributors have every incentive to encourage antimicrobial drug misuse.

**Figure:**
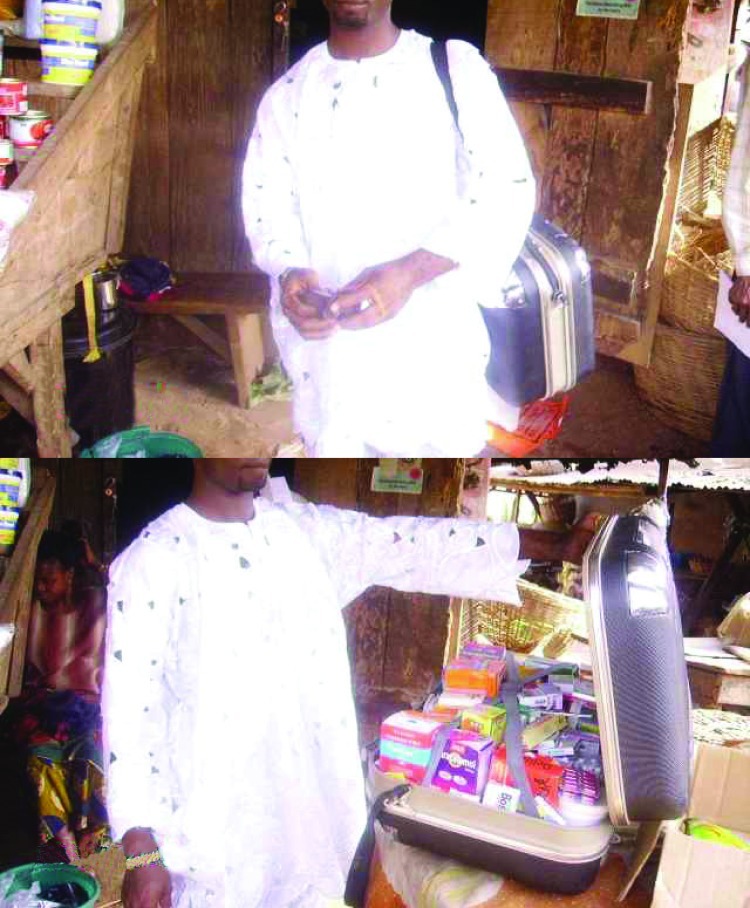
Itinerant medicine vendor in Oja-tuntun marketplace, Ile-Ife, Nigeria.

### Quality Assurance

The relative scarcity of antimicrobial drugs in poor countries with a high prevalence of infectious diseases means that the demand for antimicrobial agents exceeds their supply. This imbalance, coupled with poor purchasing power, makes sub-Saharan Africa and other developing regions a counterfeiters’ paradise. Substandard products with lower-than-stated doses promote resistance, and those containing no antimicrobial drug at all promote microbial dissemination. At least 30% of medicines sold in Africa are estimated to be counterfeit, with antimicrobial agents the most popular target (*33*). However, fake drugs are not the only poor quality pharmaceuticals on the market (*34*). Substandard drugs also include medicines that were appropriately manufactured but improperly stored. Proper storage in the tropics requires expensive electrical equipment, a constant electricity supply, pharmaceutical handling expertise, and an efficient supply chain, which do not exist in many parts of Africa. It has long been known that antibiotics are unstable at ambient tropical conditions, but shelf lives and packaging are not adapted to preserve drug potency or mark their degradation in countries where these drugs are most needed.

### Antimicrobial Drug Supply and Distribution

Prescribing health workers and their patients, particularly those who are poor, in sub-Saharan Africa continually battle a “drug is out of stock” syndrome. Rational antimicrobial drug policies, essential decision-support tools in the battle against resistance, are impossible to develop or to implement without an ensured supply of a reasonable range of drugs. Antimicrobial cycling has been piloted for antimalarial drugs in some parts of Africa, but more data are needed to gauge its effectiveness. Instituting and expanding pilot programs have been hampered by drug supply issues, particularly the difficulty in removing cheaper, resistance-compromised drugs, which are all the poor can afford, from the market (*35*). As long as demand for antimicrobial drugs exceeds their supply, uncontrolled and inadequate regimens will be the norm. To combat resistance in poor countries, antimicrobial agents will have to be made more available. For best results, when improved supply increases selective pressure, drug misuse and resistant-strain dissemination must decline. Thus, antimicrobial drugs need to be made available along with the infrastructure to monitor their utility and improve selection. Special considerations are needed to encourage patients to procure and consume a complete regimen and to ensure antimicrobial drug quality as close to the point of care as possible. These are formidable challenges because antimicrobial agents are available from unsanctioned, as well as sanctioned, providers and the former may have little or no training and unorthodox means of drug distribution.

Unrestricted access to antimicrobial drugs is perhaps the most favorable acknowledged predisposing situation for development of resistance. Ideas for promoting supervised, or at least informed, access, however, need to advance beyond proposing improved legislation because most countries already have laws proscribing unsanctioned distribution. Illegal peddling is a function of inadequate law enforcement and the reality that drug selling provides a livelihood for otherwise underemployed persons. Notably, unofficial outlets are the only source of life-saving medications for many rural residents in locations where enforcing laws modeled on wealthy societies could do more harm than good. Until the infrastructure to effectively abolish unsanctioned drug distribution is available, incorporating informal distributors into a containment strategy may be the wisest option. Focusing interventions predominantly on formal health delivery systems enhances the quality of care available to the wealthy but neglects the supply chain on which the less privileged majority are largely dependent.

### Enhanced Infectious Disease Control

The poverty–resistance cycle operates within a larger cycle of poverty and disease. Selective pressure for resistance is, in almost all cases, a response to actual or supposed infection, and resistant bacteria are largely spread through the same routes as pathogens. One of the most effective means of conserving antimicrobial drugs is therefore preventing infections. The effects of enteric infections in Africa are almost entirely driven by poor access to safe water and sanitation. Thus, once resistant pathogens emerge, they are easily spread. The commensal reservoir of resistance genes spreads through the same channels, undetected but providing a ready source of resistance genes and elements that can be transmitted to pathogens. Middle-class and affluent Africans typically reside in the few areas where piped water is available or have private water supplies. Providing safe water and sanitation to those who cannot afford these capital-intensive options and to public institutions such as schools, health centers, and markets is the single most important intervention for preventing outbreaks and sporadic cases of diarrheal disease, including those caused by resistant organisms.

Interventions that affect disease prevalence attack resistance at the root of the problem and therefore have the greatest chance of success. Effective implementations of vaccination and drug use policies, such as the World Health Organization’s Integrated Management of Childhood Illnesses, represent examples that address community-acquired infections that disproportionately affect economically disadvantaged populations (*29*). Unfortunately, even though the effectiveness of some of these interventions has been demonstrated, their access by poor populations is often not assured (*36*).

Malnourished or otherwise immunocompromised patients are more likely to have inadequate economic resources, and they become the target of resistant pathogens when these organisms are prevalent (*37*). Patients infected with resistant strains pay more for cure, lose more from extended illness (in terms of time away from work and other activities, i.e., productivity, costs of supportive therapy), and are more likely to be disabled. In wealthy countries, hospitals are often the sites where resistance emerges, and then it slowly, but eventually, seeps into the community. In poor countries, often no barrier exists between the hospital and the community. A patient’s relatives, who must be on hand to assist overstretched health systems with care, sleep under beds and in hospital corridors. In hospitals, costly infection control measures are often compromised. The potential for organisms to be transmitted into, within, and beyond the hospital is very high.

### Diagnostic Development

Diagnostic development represents a potentially powerful strategy to simultaneously improve healthcare delivery and contain resistance (*38*,*39*). The cause of bloodstream and enteric infections has diversified considerably in recent years, in part due to the definition of previously unrecognized etiologic agents but also due to the spread of HIV and the emergence of new pathogens. This increased diversity makes syndromic diagnosis of many conditions less accurate, particularly in areas where surveillance does not occur. Better systems are needed to provide laboratory support for serious cases, outbreaks, and routine surveillance. As disease control efforts begin to yield fruit, syndromic diagnosis will become increasingly inaccurate and laboratory diagnosis even more essential.

If diagnostic tests for poor patients are subsidized as well as, or better than, medicines, health professionals and patients would be more likely to use them. Additionally, sentinel laboratory facilities will also provide data on local causes of infections and prevailing susceptibility patterns to inform local prescribing and alleviate the prevailing poverty of information (*40*). Currently, studies that claim a global or worldwide coverage often exclude Africa so that as in the case of poor populations, the plight of poor countries, particularly as relevant to resistance, is underdocumented (*41*). Although programs outside Africa collate decades worth of susceptibility data, only 1 or 2 tertiary-level care centers in Nigeria and Ghana can produce complete records of susceptibility data from the past 5 years. Many African laboratories that perform susceptibility testing often cannot collate, store, or disseminate surveillance data, even though open source software is available for the purpose (*41*). Much of the data they do generate is produced with antibiotic disks donated by pharmaceutical companies with a repertoire that does not necessarily reflect the best choices for patients or even available stock. Diagnostic development could also help alleviate the pressing need for antimicrobial drug quality assurance. Precise assessment of drug content is beyond the capabilities of a basic microbiology laboratory, but rudimentary diagnostic laboratories can be equipped to identify outright counterfeits and severely degraded antimicrobial agents. Finally, the weak laboratory capacity makes it difficult to evaluate the success of interventions targeting resistance or other aspects of global disease control. Diagnostic microbiology laboratories should be considered an integral part of healthcare delivery in parts of the world where most patients visiting a health center have a microbial infection.

## Conclusion

Resistance is encountered with virtually every infectious disease. Few proven mechanisms exist for resistance control, and almost none have been validated in the developing-country setting. The dearth of intervention study data is particularly acute in the context where infectious disease prevalence is high and access to antimicrobial agents is low, which best describes the situation faced by low-income persons in Africa. Most intervention studies in developing countries have focused on relatively inexpensive and easily piloted educational interventions. Educational interventions push against the strong influence of unregulated distribution, sometimes accompanied by unscrupulous counteradvertising, and their value has not been evaluated in the long run. Importantly, although educational interventions typically yield positive results, these results are modest (*31*). Other methods may in fact be more cost effective, or might boost the value of education. Strategies that have been evaluated and found to deal with the problem of resistance need to be further diversified in poor countries. Safe water and sanitation, addressing the imbalance between antimicrobial drug supply and demand, and building realistic infrastructure for rational antimicrobial use are priority areas for resistance control that could address the short- and long-term disease effects on the poor.
